# The Effect of Nutritional Interventions on Long-Term Patient Survival in Advanced Chronic Kidney Disease

**DOI:** 10.3390/nu13020621

**Published:** 2021-02-14

**Authors:** Almudena Pérez-Torres, M. Elena González García, Marta Ossorio-González, Laura Álvarez García, M. Auxiliadora Bajo, Gloria del Peso, Ana Castillo Plaza, Rafael Selgas

**Affiliations:** 1 Nutrition Department, Hospital Universitario Santa Cristina, 28009 Madrid, Spain; almudenapereztorres@gmail.com; 2 Nephrology Department, Hospital Universitario La Paz, 28046 Madrid, Spain; marta.ossorio@salud.madrid.org (M.O.-G.); lalvarezg@salud.madrid.org (L.Á.G.); mauxiliadora.bajo@salud.madrid.org (M.A.B.); gloria.delpeso@salud.madrid.org (G.d.P.); acplaza@salud.madrid.org (A.C.P.); rafael.selgas@salud.madrid.org (R.S.)

**Keywords:** malnutrition, nutrition intervention, chronic kidney disease, survival, diet

## Abstract

Patients with end-stage kidney disease (ESKD) are at high risk of malnutrition and subsequent related mortality when starting dialysis. However, there have been few clinical studies on the effect of nutritional interventions on long-term patient survival. A 2-year longitudinal study was conducted from January 2012 to December 2016. A total of 186 patients with non-dialysis ESKD started the nutritional education program (NEP), and 169 completed it. A total of 128 patients participated in a NEP over 6 months (personalized diet, education and oral supplementation, if needed). The control group (*n* = 45) underwent no specific nutritional intervention. The hospitalization rate was significantly lower for the patients with NEP (13.7%) compared with the control patients (26.7%) (*p* = 0.004). The mortality odds ratio for the patients who did not receive NEP was 2.883 (95% CI 0.993–8.3365, *p* = 0.051). The multivariate analysis showed an independent association between mortality and age (OR, 1.103; 95% CI 1.041–1.169; *p* = 0.001) and between mortality and the female sex (OR, 3.332; 95% CI 1.054–10.535; *p* = 0.040) but not between mortality and those with NEP (*p* = 0.051). Individualized nutrition education has long-term positive effects on nutritional status, reduces hospital admissions and increases survival among patients with advanced CKD who are starting dialysis programs.

## 1. Introduction

Protein–Energy wasting (PEW) is defined by the International Society of Renal Nutrition and Metabolism (ISRNM) as ‘‘the state of decreased body pools of protein with or without fat depletion or a state of diminished functional capacity, caused at least partly by inadequate nutrient intake relative to nutrient demand and/or which is improved by nutritional repletion” [1]. Its prevalence on patients with end-stage kidney disease (ESKD) ranges from 11 to 54% [2]. Among the causes of PEW in patients with ESKD are inadequate nutrient intake, difficulties in adhering to dietary restrictions, and hypercatabolism caused by the disease itself or by comorbidities [3].

Uremic malnutrition in pre-dialysis patients is associated with increased morbidity and mortality and a greater deterioration of kidney function [4]. There are few studies, however, that assess the effect of nutritional intervention on these patients, and its prognostic value in terms of survival in ESKD has been clearly underanalyzed, despite its existence and wide acknowledgement [5,6,7]. Campbell et al. [5] performed a 4-month study on 51 ESKD patients (27 in a control group and 24 in an NEP) and found that in the control group, the proportion of patients with malnutrition increased from 11% to 22%, whereas all the malnourished patients in the intervention group (20.8%) reversed their situation. Cliffe et al. [6] studied 11 ESKD patients and found that, with dietetic intervention, it was possible to maintain or improve nutritional status in these patients.

The National Kidney Foundation Kidney Disease Outcomes Quality Initiative guidelines and ISRNM expert committee recommend nutritional interventions during therapy for pre-dialysis patients [8,9,10].

We hypothesized that nutritional interventions for patients with ESKD, defined as a nutritional education program (NEP), might have general positive long-term (2 years) effects, without kidney function deterioration.

The aim of this study is to evaluate the long-term effects of a nutritional intervention in the pre-dialysis stage on health results, survival and comorbidities during the following 2-year period among patients with advanced CKD. To confirm the immediate efficacy of this intervention, we also tested the effects of a NEP on morbidity and mortality and compared the results at 6 months between patients with and without nutritional support.

## 2. Materials and Methods

### 2.1. Study Participants 

**Figure 1:**
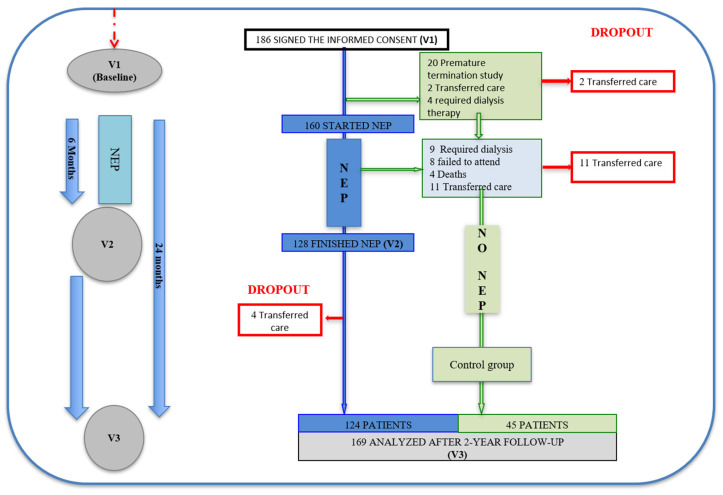
Patient flow diagram, indicating the selection and discontinuation, according to groups. Abbreviation: NEP, Nutrition Education Program.

The initial number of participants was 186, and 169 completed the final evaluation (2 years later). A total of 160 of these patients were included in the NEP (Figure 1) from March 2008 to September 2016 and 128 finished it. 

The inclusion criteria were advanced kidney disease, defined as a creatinine clearance <20 mL/min/1.73 m2 (stages 4 and 5, not undergoing dialysis [20 patients had creatinine clearances <30 mL/min/1.73 m2]); 18 years of age or older; no cognitive impairment; and having signed the informed consent. The exclusion criteria were active neoplasia, infection, severe lung disease, and hospitalization during the study period.

The study was approved by the ethics committee of Hospital Universitario La Paz and was conducted according to the guidelines of the Declaration of Helsinki. The patients signed a written informed consent document prior to inclusion. After 6 months of the NEP, we collected data to assess the efficacy and safety of the intervention. Patients were evaluated at baseline and at 6 and 24 months after beginning the NEP. All patients started dialysis as indicated based on their individual clinical and biochemical parameters.

### 2.2. Study Design

We conducted a 2-year longitudinal prospective study with 169 patients, 128 of whom participated in an NEP during the first 6 months of the study. The full study population was selected from patients in the advanced kidney disease care program at the Nephrology Department of University Hospital La Paz in Madrid, Spain.

### 2.3. Nutrition Education Program

Selected patients were included in an NEP, which consisted of an individualized diet plan based on the patient’s initial nutritional status, with 4 nutrition education sessions, a nutritional assessment and monitoring for 6 months.

The intervention was administered by a single dietitian, aimed at providing a personalized dietary prescription (including energy [25–35 kcal/kg/day] and protein [0.75–1.0 g/kg/day] based on K/DOQI guidelines). [8]

In the nutrition education sessions, the patients were addressed over protein and energy intake, content of phosphorus and potassium in foods, cooking techniques, and a fourth issue chosen according to the patient’s specific needs; for example, content of fat, cholesterol, or sucrose in foods. We also elaborated a dietary plan after obtaining information from a 3-day dietary record. We used photographic albums as material support to estimate portion sizes or to explain to patients how to read and understand food labels. 

Thirty-two (25%) patients required specific nutritional support (oral nutritional supplementation [ONS]), those who did not meet the energy and protein requirements proposed for CKD patients (CKD 4–5 no d) by the K/DOQI guidelines [8] with PEN exclusively. Thirty patients took ONS specific for patients with CKD (high in calories (2.0 Kcal/mL.) and low in protein (8–10 gr serving)), and two patients took specific ONS for diabetics (1.0–1.5 Kcal/mL and 10–12 gr protein/serving).

During the program, the patients continued with their usual medical treatment. We collected clinical data at the start of the NEP and during the follow-up.

### 2.4. Laboratory Parameters

Pre-prandial blood samples were obtained to determine albumin, prealbumin, creatinine and creatinine clearance (with 24 h urine collection), potassium, phosphate, C-reactive protein, total cholesterol, low-density lipoprotein cholesterol (LDL-c), high-density lipoprotein cholesterol and triglyceride levels, as well as total lymphocyte counts. The patients’ diuresis volume and 24 h urine protein were also quantified. The analysis of the biochemical parameters was performed according to the standardized method of the Biochemistry Laboratory of University Hospital La Paz.

Normalized protein nitrogen was calculated using the formula proposed by the US National Kidney Foundation ((g/kg/day) = [6.49 × urine urea nitrogen (g/day) + 0.294 (g/L/day) × vol (L)]/[vol (L)]/0.58 (L/kg)]). 

### 2.5. Anthropometrics and Body Composition

Anthropometric measurements were performed using standard techniques following international guidelines (World Health Organization, 1976) [11] 6 and were taken with the patients in their underwear and bare-footed. Body weight was measured using a single-frequency body composition analyzer (TANITA BC-420MA, Biológica Tecnología Médica S.L. Barcelona, Spain). Height was measured using a measuring rod (range, 80–200 cm), and mid-arm circumference (MAC) was measured using a stretchable measuring tape. The tricipital skinfold (TSF) was measured using a Holtain caliper with a jaw width of 20 cm and sensitivity of 0.2. The mid-arm muscle circumference (MAMC) was calculated in centimeters as follows: MAMC = MAC − (3.14 × TSF). Body mass index (BMI) was calculated based on the weight and height measurements (weight [kg]/height2). The body composition measurements included a bioimpedance analysis (BIA-101, Akern Systems, Florence, Italy) at 50 kHz. Body composition parameters were analyzed and adjusted by sex.

### 2.6. Dietary Intake

Each patient’s overall dietary intake was recorded on a food intake record for 3 consecutive days, listing all food intake (including hydration); one of these days was on a weekend. The food intake records were double-checked with the patients. The diet’s caloric and nutritional value was quantified using DietSOURCE^®^3.0 nutritional software (Novartis Medical nutrition, Barcelona, Spain). 

### 2.7. Nutritional Assessment

The nutritional assessment was performed according to the protein–energy wasting (PEW) criteria proposed by the ISRNM [1]. To determine the nutritional status according to ISRNM criteria, the patient had to meet 1 criterion in 3 of the 4 categories that determined the presence of PEW over 2 months:

- Biochemical category: albumin <3.8 g/dL; prealbumin <30 mg/dL body mass; cholesterol <100 mg/dL without lipid-lowering medication (in our case, this criterion was not employed, given that 147 patients consumed such medication).
- Body mass category: BMI <23 kg/m^2^; unintentional 5% weight loss over the last 3 months or 10% weight loss over the last 6 months.
- Muscle mass category: loss of 10% of MAMC in relation to the 50th percentile.
- Intake category: protein catabolism rate (normalized protein nitrogen) <0.6 g/kg/day; energy intake <25 kcal/kg adjusted to weight/day.

### 2.8. Number of Hospital Admissions

We recorded the number, length and causes of hospitalizations during the 2-year follow-up (both planned and emergency hospital stays), excluding hospitalizations shorter than 4 days and those related to kidney disease management, such as transplantation and dialysis (fistula/catheter).

### 2.9. Mortality

Mortality and its causes were also recorded.

### 2.10. Statistical Analysis

The qualitative variables are presented as absolute frequencies and percentages, while the quantitative variables are presented as means and standard deviations (SD).

The qualitative variables were compared between the two groups using the chi-squared test or Fisher’s exact test, depending on the data distribution. To compare the patients’ differences between the start and end of the program, we employed the Wilcoxon (quantitative variables) and McNemar tests (qualitative variables). The quantitative variables between the two groups were compared using the Mann–Whitney U test or Student’s t-test, depending on the data distribution.

To determine the patients’ profiles, we performed a multivariate step logistic regression model using the conditional forward step method. The results of the model adjustment are presented as odds ratio (OR), their corresponding 95% CI and the *p*-values.

Survival during the 2-year follow-up was calculated using the Kaplan–Meier method; for the survival comparison between the groups, we employed the log rank test. Survival times are presented as means and 95% confidence interval.

All statistical tests were bilateral, with a significance level of 0.05. The statistical analysis was performed using Statistical Package for the Social Sciences, version 17.0 (SPSS Inc., Chicago, IL, USA).

## 3. Results

### 3.1. General Characteristics of the Population

A total of 169 patients (89 men, 52.7%) with a mean age of 66.1 ± 15.9 years completed the study. The primary CKD etiology was diabetes mellitus in 71 (42.0%) patients, 6 (3.5%) of whom had type I diabetes. The other etiologies were nephroangiosclerosis (23 patients; 13.6%), glomerulonephritis (22 patients; 13%), polycystic kidney disease (19 patients; 11.2%), unknown (17 patients; 10%) and other (17 patients; 10%). 

The main comorbidities in the study patients were congestive heart failure or unresolved ischemia (52 patients; 30.8%), peripheral arterial disease (39 patients; 23.1%), functionally severe physical sequelae (9 patients; 5.3%), chronic obstructive pulmonary disease (15 patients; 8.9%) and stroke (15 patients; 8.9%). We found no differences by sex, etiology or comorbidity between the NEP and control group.

### 3.2. Results of the Nutritional Education Program at 6 Months

A total of 128 patients underwent the NEP, and 32 (25%) patients required specific nutritional support (ONS). We found no differences by sex or age between the two groups. Table 1 shows the follow-up for the biochemical, anthropometric and body composition parameters between the patients who required ONS or not.

The NEP+ONS group had the poorest nutritional status at the start, with lower prealbumin levels, weight and phase angle in the bioimpedance study. In the NEP group, the serum potassium and phosphorus levels decreased, and the lipid profile improved over the observation. 

The BMI increased in the NEP+ONS group, with a decrease in total body water and an increase in intracellular water and fat mass. The BMI decreased in the NEP group, but muscle mass increased while total body water decreased.

We found significant differences in the response to the nutritional intervention between the men and women. The men improved their nutritional status significantly, with only 4 (6.5%) out of 18 (29.5%) men remaining in the PEW state. In contrast, 10 (14.9%) of the 17 (25.4%) women remained in the PEW state (*p* = 0.024). In fact, the nutritional state worsened in 3 of these women after the intervention. 

**Figure 2:**
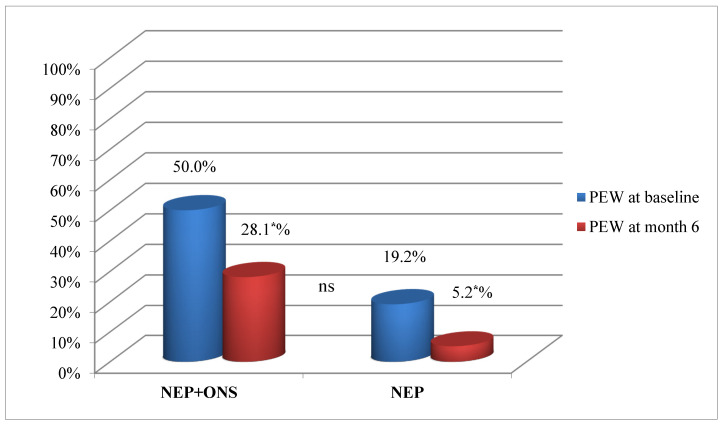
Protein–energy wasting progression for nutritional educational program plus oral supplementation group and the nutritional educational program only group. Percentage of participants, NEP+ONS or only NEP with PEW at baseline and at the end of the intervention. Abbreviations: NEP, nutrition educational program; ONS, oral nutrition supplementation; PEW, protein energy wasting. *p* was calculated with the chi-squared test. (*) Statistically significant difference (*p* < 0.05).

At the start of the NEP, the number of patients with PEW was significantly higher in the NEP+ONS group, decreasing significantly in both groups at 6 months (Figure 2).

### 3.3. Comorbidities and Survival at the 2-Year Follow-Up

Of the 169 patients who completed the 2-year follow-up, 124 (73.4%) underwent the NEP and 45 (26.6%) did not. Table 2 shows the demographic characteristics and follow-up of the biochemical parameters of the NEP and control groups. Albumin levels and normalized protein catabolic rates increased significantly in the NEP group, with no differences in the renal replacement therapy technique, comorbidities or anthropometric parameters between the two groups at the 2-year follow-up.

Hospitalization causes were 42.1% (8) due to infections, 31.6% (6) due to cardiovascular disease, and 26.3% (5) from other causes. The hospitalization rate was significantly higher for the control group than for the NEP group (26.7% [12 patients] vs. 13.7% [17 patients], respectively; *p* = 0.004). The mean hospital stay was also shorter for the NEP group, but the difference was not statistically significant (8.6 ± 3.3 vs. 9.9 ± 5.4 days for NEP and control, respectively; *p* = 0.183).

BMI had a weak association with the number of hospitalizations during the 2-year follow-up, and there were no differences in the other study variables.

**Figure 3:**
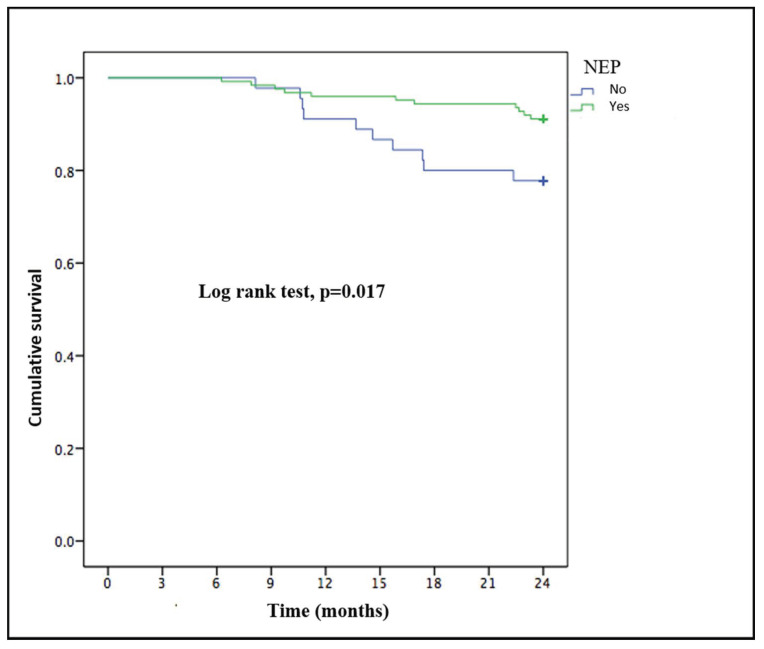
Kaplan–Meier survival curve of patients with end-stage renal disease who underwent or not a personalized nutritional education program.

Twenty-one patients (12.4%) died during the 2-year follow-up: 6 (28.6%) due to infections, six (28.6%) due to cardiovascular disease, three (14.3%) due to tumors, four (19%) from treatment withdrawal and two (9.5%) from other causes. There were no differences between the NEP and control groups in terms of cause of death. The patients who died were more likely to be older (77.5 ± 8.2 vs. 64.5 ± 16.1 years; *p* < 0.001), women (18% (16) vs. 6.3% (5); *p* = 0.021) than those who survived and not undergoing NEP (22.2% (10) vs. 8.9% (11); *p* = 0.024). These variables maintained their statistically significant relationship to mortality in the multivariate analysis, except for NEP (*p* = 0.051) (Table 3). There were no differences in the renal treatment modality between the patients who died and those who survived. The NEP group had longer survival than the control group after applying the Kaplan–Meier method (*p* = 0.017) (Figure 3).

## 4. Discussion

Our study’s main finding is that a nutritional intervention in phase IV-V of non-dialysis CKD has long-term effects on survival and morbidity (number of hospitalizations), although this effect did not reach statistical significance among women, and these effects occurred regardless of the type of renal replacement therapy started during follow-up, providing an added value to the nutritional intervention in the pre-dialysis stage.

### 4.1. Nutritional Education Program Efficacy at 6 Months

Our data show that the individualized nutritional intervention, with or without ONS, is effective and safe for patients with non-dialysis CKD, decreases the number of patients who developed PEW and produces no renal function impairment.

A few studies have evaluated the effect of nutritional intervention prior to commencing dialysis [12], but they had a smaller sample than ours. Campbell et al. [5] conducted a 4-month study with a control group of 27 patients (who received only written instructions) and an intervention group of 24 patients (who received individual nutritional education). The intervention group managed to achieve improved body mass and nutritional status, as measured by the Subjective Global Assessment, and increased their food intake. Although our data agree with these results, the study by Campbell et al. provided no information on ONS. In our study, all the patients who underwent individual NEP also underwent ONS if needed. 

Most of the studies we consulted showed that nutritional status improves with ONS in patients undergoing dialysis, as represented by increased serum albumin and prealbumin levels [13,14,15]. Sezer et al. [16] compared 32 hemodialysis patients with ONS and 30 patients who received only individual nutritional education during a 6-month follow-up. Serum albumin levels increased only in those patients from the nutritional education group, unlike in our study, where although both groups improved, only the group that underwent ONS achieved truly significant changes. This finding could also be due to the initial lower albumin levels at the start of the nutritional intervention for the ONS group.

We found only one study on dialysis in a Spanish population by Hernández-Morante, which compared the results of NEP versus ONS and concluded, like ours, that both treatments are equally effective [17]. The main difference between the two studies is that in the study by Hernández-Morante, the patients were randomly assigned to a group, and the ONS group received no dietary advice.

Due to the effects of PEW on mortality, the ISRNM nutritional guidelines recommend nutritional advice as the first step for PEW treatment in dialysis and pre-dialysis patients and specific oral nutritional supplementation thereafter [9,18]. All of these measures were included in our individualized NEP. We found no study that implemented and evaluated the ISRNM recommendations in populations with non-dialysis CKD.

### 4.2. Long-Term Effects of Nutritional Education Programs on Health Outcomes 

The increase in hospitalization rates is one of the main consequences of malnutrition, leading to increased health expenditures [19,20]; however, only the FINES study, which was conducted with a population undergoing dialysis, showed that oral supplementation improved serum albumin and prealbumin levels and hospitalization rates in dialysis patients [17]. We found no study that evaluated these data in pre-dialysis patients; our study is, therefore, the first that we know of to link lower hospitalization rates to individualized nutritional interventions. 

Kovesdy et al. [4] assessed the ratio of serum albumin levels, total lymphocyte counts and white cell counts to pre-dialysis mortality over a 3.3-year follow-up, with a patient sample with similar characteristics to our study population. The authors found an independent association between these parameters and mortality (unrelated to sex) but did not provide information on the dialysis technique that the participants were undergoing at the end of the follow-up. The number of deaths (36.1%) was higher than in our study (12.4%). A possible explanation is that the patients were not undergoing individualized nutritional treatment in the Kovesdy et al. series.

Despite the limitations of serum albumin as a marker of malnutrition, we consider, as do Mazairac et al. [21,22], that albumin alone reflects the mortality risk in hemodialysis populations similarly to several combined nutritional assessment methods. Serum albumin is one of the main methods for assessing nutritional status in patients with ESKD.

We agree with other studies that the dialysis technique does not affect the mortality or hospital admission rate [23,24] and that age is an independent predictor of mortality [25].

Recent studies have indicated the need to consider patient sex so as to individualize the care during CKD [26,27]. Based on our results, we agree with this conclusion; women fare far worse with nutritional interventions, and these effects remain after the 2-year follow-up. One possible factor in this relationship could be the higher incidence of sarcopenic obesity among our female patients (more obesity than in men), although we do not have data on the prevalence of sarcopenic obesity. Obesity can make the nutritional intervention more difficult because it can mask the malnutrition, and there is a lack of clinical guidelines for this condition. 

Our study’s main limitation is not having designed a nutritional intervention differentiated by sex beforehand, due to a lack of information in the literature. It is possible that a larger sample size could confirm the independent relationship between female sex and mortality. Although we analyzed all categories related to nutritional status, a complete nutritional analysis could not be performed at the end of the study.

## 5. Conclusions

Our study shows that a nutritional intervention is useful in non-dialysis CKD, and its effects last into the latter stages of CKD (dialysis). Nutritional therapy should be individualized according to sex and age, and the population with obesity is at increased risk of receiving a nutritional misdiagnosis.

## Figures and Tables

**Table 1:** Biochemical, anthropometric and body composition parameters for the nutritional educational program plus oral supplementation group and the nutritional educational program only group.

	NEP+ONS			NEP		
	Month 0	Month 6	*p*	Month 0	Month 6	*p*
**Age**	70.5 ± 14.5			66.0 ± 14.8		
**Albumin, g/dL**	3.3 ± 0.5 *	3.5 ± 0.5	0.106	3.6 ± 0.4	3.8 ± 0.3	<0.05
**Prealbumin, mg/dL**	26.0 ± 5.6 *	**30.0 ± 4.4 ^b^**	<0.05	**31.1 ± 6.4**	**32.1 ± 6.2 ^b^**	0.480
**CrCl, ml/min**	16.8 ± 4.1	18.9 ± 6.2	<0.05	17.6 ± 3.8	19.5 ± 6.5	<0.05
**Creatinine, mg/dL**	3.4 ± 1.1	3.3 ± 1.0	0.252	3,8 ± 1,2	3.6 ± 1.3	0.400
**Proteinuria, g/24**	2.0 ± 3.1	1.8 ± 2.8	0.086	1.7 ± 1.6	1.6 ± 1.7	0.176
**Potassium, meq/L**	4.8 ± 0.6	4.6 ± 0.5	0.105	4.8 ± 0.6	4.6 ± 0.5	<0.05
**Phosphorous, mg/dL**	3.8 ± 0.9	3.8 ± 0.6	0.592	4.1 ± 0.9	3.9 ± 0.7	<0.05
**LDL, mg/dL**	109.1 ± 29.2	106 ± 30.3	0.411	114.0 ± 35.2	104.9 ± 28.1	<0.05
**Triglycerides, mg/dL**	125.3 ± 53.7	120.2 ± 48.4	0.362	141.5 ± 61.6	125.6 ± 38.8	<0.05
**Weight, kg**	61.5 ± 13.1 *	**62.2 ± 11.3 ^b^**	0.590	**73.9 ± 12.4**	**72.2 ± 11.3 ^b^**	<0.001
**Height, m**	160.7 ± 9.8			159.1 ± 8.9		
**BMI, kg/m^2^**	24.5 ± 5.4 *	**24.7 ± 4.6 ^b^**	<0.05	**28.8 ± 4.4**	**28.0 ± 4.0 ^b^**	<0.001
**TSF, mm**	15.3 ± 7.1 *	**15.5 ± 7.1 ^b^**	0.091	**20.1 ± 7.1**	**19.7 ± 7.0 ^b^**	<0.001
**MAMC, mm^2^**	20.5 ± 3.5 *	21.0 ± 3.4	<0.05	23.9 ± 3.9	23.9 ± 3.6	0.956
**Resistance, ohms**	**550.4 ± 99.9**	**535.3 ± 96.9 ^b^**	0.061	**507.3 ± 74.7**	**506.4 ± 79.9 ^b^**	0.073
**Reactance, ohms**	**33.4 ± 7.7**	**41.7 ± 8.5 ^b^**	0.053	**44.8 ± 10.9**	**46.3 ± 10.7 ^b^**	0.073
**Phase angle, degrees**	**3.9 ± 0.9**	**4.5 ± 0.8 ^b^**	0.051	**5.2 ± 1.2**	**5.21 ± 0.6 ^b^**	0.540
**Na/K Exchange**	**1.6 ± 0.5**	**1.4 ± 0.5 ^b^**	0.059	**1.0 ± 0.2**	**1.0 ± 0.5 ^b^**	0.974
**TBW, %**	**57.7 ± 6.0**	**54.6 ± 9.6 ^b^**	0.054	**52.5 ± 6.1**	**52.6 ± 6.0 ^b^**	0.608
**ECW, %**	**58.7 ± 6.2**	**53.6 ± 4.4 ^b^**	<0.05	**50.5 ± 5.8**	**49.5 ± 5.5 ^b^**	<0.05
**ICW, %**	**41.1 ± 6.2**	**46.4 ± 4.4 ^b^**	<0.05	**49.4 ± 5.8**	**50.6 ± 5.5 ^b^**	0.070
**Fat mass, %**	**27.7 ± 12.5**	**30.8 ± 11.9 ^b^**	<0.05	**33.4 ± 8.4**	**32.7 ± 8.8 ^b^**	0.634
**Muscle mass, %**	39 ± 13.6	40.2 ± 12.7	0.084	38.5 ± 7.6	40.4 ± 7.5	<0.05
**Body cellular mass index**	7.0 ± 2.6	7.7 ± 2.7	<0.05	8.2 ± 2.1	8.5 ± 1.8	<0.05

Abbreviations: BMI, body mass index; CrCl, creatinine clearance; ECW, extracellular water; LDL-c, low-density cholesterol; ICW, intracellular water; MAMC, mid-arm muscle circumference; NEP, nutritional educational program; ONS, oral supplementation; TBW, total body water; TSF, tricipital skinfold. Values are presented as mean ± standard error of the mean. *p* was calculated by Wilcoxon-test. (*) Differences at baseline between NEP+ONS patients and NEP patients, *p* was calculated by student *t*-test. (b) Differences in the intervention at 6 months between the two studied groups. Significant differences are highlighted in bold.

**Table 2:** Demographic characteristics and clinical outcomes between the nutritional education program group and control group after the 2-year follow-up.

	NEP group *N* = 124 (73.4%)			Control Group *N* = 45 (26.6%)		
	Month 0	2 Years		Month 0	2 Years	
**Age, years**	67.2 ± 14.9			63.2±18.3		**0.036**
**Male sex, n (%)**	57 (46%)			32 (71.1%)		**0.050**
**Albumin, g/dL**	3.5 ± 0.4	3.8 ± 0.5	**<0.001**	3.6±0.4	3.4 ± 0.5	**0.027**
**nPNA, g/kg/day**	1.18 ± 0.35	1.30 ± 0.27	**0.020**	1.2±0.35	1.3 ± 0.34	0.617
**Weight, kg**	70.5 ± 13.3 *	70.1 ± 11.6	0.084	74.0±18.2^*^	75.0 ± 20.6	0.635
**BMI, kg/m^2^**	27.55 ± 4.9	27.5 ± 4.4	0.110	27.0±5.3	27.1 ± 6.18	0.635

Abbreviations: BMI, body mass index; NEP, Nutritional Education Program; nPNA, normalized nitrogen appearance. Values are presented as mean ± standard error. P was calculated with the Wilcoxon test. Significant differences are highlighted in bold. (*) Differences at baseline between NEP+ONS patients and NEP patients. *p* was calculated with Student’s *t*-test.

**Table 3:** Multivariate analysis for mortality.

		Multivariate Model			
		*N*	OR	95% CI	*p*
**Age**		169	1.094	1.035–1.157	**0.001**
**Sex**	Male	80			
	Female	89	3.332	1.054–10.535	**0.040**
**NEP**	Yes	124			
	No	45	2.883	0.993–8.365	0.051

Abbreviations: NEP, Nutritional Education Program; OR, odds ratio. Significant differences are highlighted in bold.

## Data Availability

The data presented in this study are available on request from the corresponding author.
